# Inactivation of *Salmonella typhimurium* SL1344 by Chlorogenic Acid and the Impairment of Cellular Integrity

**DOI:** 10.3389/fmicb.2022.887950

**Published:** 2022-04-14

**Authors:** Liang Yang, Chunlin Zhang, Zijing Su, Liang Zhao, Jiaxin Wu, Xiaoying Sun, Xiujuan Zhang, Xiaoqing Hu

**Affiliations:** ^1^Department of Brewing Engineering, Moutai Institute, Renhuai, China; ^2^School of Biotechnology, Jiangnan University, Wuxi, China; ^3^State Key Laboratory of Food Science and Technology, Wuxi, China; ^4^Joint Laboratory on Food Safety, Jiangnan University, Wuxi, China

**Keywords:** chlorogenic acid, bactericidal effect, *Salmonella* *typhimurium*, *Eucommia ulmoides*, impairment of cellular integrity, cellular morphology

## Abstract

Chlorogenic acid (CGA) is an antibacterial agent that can be isolated from *Eucommia ulmoides* Oliver, a Chinese medicinal and edible plant food. The inhibitory effect of CGA on bacterial growth and stiffness of the outer membrane (OM) had been reported, while more evidence were required to elucidate its impairment of cell wall. In this study, the morphological and physiochemical changes of *Salmonella* cells under CGA treatment were investigated. Firstly, the minimum inhibitory concentration (MIC) of CGA against *Salmonella* was assayed. Later, the permeability of OM and activity of the proteins released were measured and observed to reveal the alteration of OM characteristic and cellular morphology. Finally, reactive oxygen species and cell membrane fluidity were analyzed, respectively, to elucidate how CGA damaged cell surface. The results showed that MIC of CGA against *Salmonella* was 6.25 mg/L. Under sub-lethal doses of CGA, the OM permeability and the release of soluble proteins were enhanced evidently, and *Salmonella* cells showed more deformed and shrunken, confirming the impairment of cellular integrity under CGA. Finally, the possible cause of cell surface damage was investigated. the fluidity of the membrane was increased upon CGA treatment, which may the possible cause of OM by CGA.

## Introduction

*Eucommia ulmoides* Oliver (EU) is a rare Chinese medicinal and edible plant food. Its leaves and bark have been used as medicine for thousands of years. The leaves of EU contain a variety of biologically active ingredients, such as iridoids, phenylpropanoids, flavonoids, and other substances (He et al., 2014). Among these bioactive substances, chlorogenic acid (CGA) is a phenylalanine-containing compound and considered as the main active ingredient in the leaves of EU (Chao et al., 2009). CGA has substantial pharmacological effects, such as antibacterial activity (Kremr et al., 2016), and has attracted widespread attention (Klaric et al., 2020; Lu et al., 2020). Therefore, a variety of techniques have been developed for the extraction and purification (Liu et al., 2016a,b).

Nowadays, the antibacterial effects of plant-derived CGA against bacteria have been reported. The minimum inhibition concentration (MIC) value of CGA against the bacterium *Bacillus subtilis* (*B. subtilis*) was 0.3125 mg/L (Wu et al., 2018a), and the inhibition zone for *Salmonella lignieres* on PDA medium is 10–14 mm in diameter (Chen et al., 2010). However, the bactericidal mechanisms of CGA were unclear. Ren et al. (2015) reported that CGA extracted from honeysuckle could inhibit the growth of *E. coli* by affecting the stiffness of the cell wall (i.e., the outer membrane, OM). CGA extracted from *Arctium lappa L.* increased the permeability of the plasma membranes of cells of *Staphylococcus aureus*, with an MIC value of 0.04 mg/L, thus inhibiting the synthesis of the bacterial cell envelope (Lou et al., 2011).

However, there was almost no systematic investigation of the cell wall, such as morphological, physiological and biochemical characteristics after CGA treatment, which were crucial for discovering the mechanism underlying bactericidal effect of CGA. Therefore, the important food-borne pathogen *Salmonella* was employed in the current work and the influences of sub-MIC CGA on cellular integrity were studied for the first time. As one of the most popular pathogens that can be isolated from egg, meat and humans, *Salmonella* pollution and infection resulted in major problem for the food industry and public health hazard worldwide, thus it is of significance to explore green and safe bactericidal chemicals.

In the previous reports on cell wall damage-based inactivation of bacteria (Hu et al., 2018; Wu et al., 2018b; Chu et al., 2019), there existed a close relationship between the integrity of cell surface and characteristics of OM, such as the permeability and leakage. Thus in the present study, more evidence on the permeability and fluidity of OM, release of the intracellular soluble proteins, and reactive oxygen species (ROS) were achieved, and the present study will lay a foundation for revealing the antibacterial mechanism of CGA and EU food development.

## Materials and Methods

### Bacterial Strain and Culture Conditions

*Salmonella typhimurium* SL1344 was cultured on LB agar plates (10 g/L tryptone, 5 g/L yeast extract, 10 g/L NaCl, and 15 g/L agar, pH at 7.0) for 24 h. Later, a single colony was picked and incubated in 50 mL LB medium without agar at 200 rpm for 8–12 h, until the optical density at 600 nm (OD_600_) reached 1.0. Both incubations were carried out at 37°C.

### Assay of Minimum Inhibitory Concentration

The Minimum Inhibitory Concentration (MIC) value of CGA against *Salmonella* was determined according to the agar dilution method (Liu et al., 2012, 2017; Fu et al., 2016), with minor modifications as follows: (1) 100 μL of LB liquid culture medium was added into the 1st to 10th wells of a 96-well microplate, and then 100 μL CGA solution (0.1 g/L) extracted from the leaves of EU was added to the 1st well and mixed well; (2) 100 μL of the mixed solution from the 1st well was pipetted and added into the next well, and then a double dilution was performed in turn. After mixing in the 10th well, 100 μL of the mixed solution was discarded; (3) Bacterial suspension was collected (OD_600_ = 0.5), and 100 μL was added into each well from the 1st to the 10th, and then mixed well. At that time, the bacterial concentration per well was 5 × 10^5^ CFU/mL; (4) 200 μL of LB liquid medium and sterile water were pipetted into the 11th and 12th wells, respectively, as blank controls; (5) The 96-well microplate was incubated in a 37°C constant temperature incubator for 24 h and OD_600_ was measured. The lowest concentration of CGA without bacterial growth after 24 h was recorded as the MIC.

### Detection of Permeability of Outer Membrane

Bacterial cells were mixed with 0.25 × MIC, 0.5 × MIC, 1 × MIC, and 2 × MIC of CGA for 8 h, harvested by centrifugation at 12,000 rpm for 5 min, then washed by 20 mmol/L phosphate buffer solution (PBS, pH 7.4) for three times, and re-suspended in PBS. Later, the permeability of OM was assayed according to a method based on the hydrophobic probe *N*-phenyl naphthylamine (NPN). Cells were incubated with NPN for 15 min at 25°C in the dark. Damaged cells were detected using a microplate reader (Synergy H1 microplate reader, Biotek-Agilent, Winooski, VT, United States), with excitation at 350 nm and emission at 420 nm (Gogry et al., 2022).

### Assay of the Extracellular Soluble Proteins

A large amount of the intracellular soluble proteins will leak outside when the intact cell wall is impaired, thus the content of extracellular soluble proteins can be regarded as an indicator of cell surface damage. The intracellular proteins were extracted and assayed based on the procedure reported by Yao et al. (2014), with minor modifications as follows: (1) 0.5 mL of *Salmonella* culture broth with logarithmic growth were inoculated into 500 mL shaking flask containing 50 mL LB liquid medium, and an appropriate amount of CGA mother liquor was added so that the final mass concentrations of CGA were obtained at 0 × MIC, 0.5 × MIC, and 1 × MIC, respectively; (2) The shaking flasks were cultured in the incubator at 37°C and 100 r/min; (3) After different incubation times (0, 2, 4, 6, 8, and 10 h), 2 mL of the culture medium was sampled and centrifuged at 4,000 rpm for 10 min; (4) 0.1 mL of the supernatant was collected, and mixed with 0.5 mL color-substrate solution. After shaking and mixing, 1 mL distilled water was further added and mixed for 2 min; (5) The absorbance was measured at 595 nm. The soluble protein content was assayed with the Coomassie brilliant blue method (Bradford, 1976), and calculated on the basis of the standard curve, with minor modifications as follows. After construction of a linear standard curve for bovine serum albumin, the measured absorbance of the sample was compared with the standard curve to calculate the soluble protein of the sample. The standard curve prepared in this experiment was *y* = 0.5983*x* − 0.1194; *R*^2^ = 0.9991.

### Alkaline Phosphatase Assay

Alkaline Phosphatase (AKP) located at the space between OM and inner membrane (i.e., plasmic membrane) in the intact cell, while it came out when OM was impaired, thus AKP activity in the environment was usually employed to assess the extent of OM damage. Referring to a method reported by Zheng et al. (2016), AKP activity was detected with some modifications as follows: (1) *Salmonella* cells with logarithmic growing were inoculated at 1% (V/V) into 50 mL LB liquid medium supplemented with CGA, and the final dose of CGA were kept at 0 × MIC, 0.5 × MIC, and 1 × MIC, respectively, (2) The shaking flasks were placed in an incubator (37°C, 100 r/min); (3) 2 mL of the culture broth was sampled every 4 h and centrifuged at 12,000 r/min for 10 min; (4) AKP activity in the supernatant was measured using AKP Assay Kit (Beyotime, P3021, China).

### Scanning Electron Microscopy Observation

*Salmonella* cells were treated by 0 × MIC, 0.25 × MIC, and 0.5 × MIC CGA for 8 h, then the cells were collected by centrifugation for 5 min at 12,000 rpm, later washed for three times with PBS (pH 7.4), and finally re-suspended in PBS containing 2.5% glutaraldehyde for 12 h. The cells were further dehydrated in water–alcohol solutions at 70% alcohol concentration for 10 min. Finally, the samples were fixed on SEM support and sputter-coated with gold under vacuum, and finally subjected to microscopic examination with SEM (Cao et al., 2019).

### Assays of Reactive Oxygen Species

Reactive oxygen species (ROS) within *Salmonella* cells treated by CGA was extracted and determined as follows: (1) A fluorescent probe 2′,7′-dichlorofluorescin diacetate (DCFH-DA) was used to react with ROS to form fluorescent DCF, which was detected with a spectrofluorophotometer with an excitation wavelength at 485 nm and an emission wavelength at 525 nm, respectively; (2) The cells suspension was incubated with different doses of CGA to obtain the final concentrations at 0 × MIC, 0.25 × MIC, and 0.5 × MIC CGA, respectively. The CGA-treated cells were harvested by centrifugation and then washed by PBS as described above; (3) The resultant cells were diluted by PBS to final cell density at 2 × 10^8^ CFU/mL, and then the cells suspension was incubated with DCFH-DA (10 mmol/L) at room temperature for 20 min in a dark room; (4) The mixture was centrifuged immediately at 12,000 rpm for 5 min at 4°C, and the bacterial precipitate was washed twice with PBS at 4°C to remove the DCFH-DA that had not entered the cell but was only attached to the cell surface; (5) ROS level was determined with a spectrofluorophotometer according to the protocol of the ROS assay kit (kit #S0033 Beyotime Biotech, China).

### Fluidity of Bacterial Cell Membranes

On the basis of the previous method (Furusawa et al., 2006), the fluidity of cell membranes were assayed with minor modifications as follows: (1) 10 mL *Salmonella* cells suspensions was mixed with different doses of CGA (0 × MIC, 0.25 × MIC, and 0.5 × MIC CGA) for 8 h; (2) The cells were harvested by centrifugation, then the bacterial cells were washed by sterilized deionized water and resuspended to a final density at OD600 = 0.8; (3) 5 μL newly prepared dimethyl sulfoxide (DMSO) with 100 μmol⋅L^–1^ 1,6-diphenyl-1,3,5-hexatriene (DPH) was added to the cells suspensions; 4) After the bacterial cells were stained for 30 min, DPH-labeled bacteria were obtained in a dark room at 25°C. The degree of fluorescence polarization (P) was measured with a fluorescence spectrophotometer with a polarization device. Detection conditions were as follows: excitation wavelength Ex = 360 nm; emission wavelength Ex = 427 nm; excitation slit 5 nm; emission slit 10 nm (Furusawa et al., 2006).

### Statistical Analysis

For the experiments mentioned-above, three independent trials were performed to obtain the mean levels. The values are expressed as mean ± standard deviation. Differences in the values of the untreated control and the samples treated by different doses of CGA were compared for statistical significance with one-way ANOVA, and a significant difference was considered to be indicated by a *p* value of less than 0.05.

## Results

### Minimum Inhibitory Concentration of Chlorogenic Acid

**FIGURE 1:**
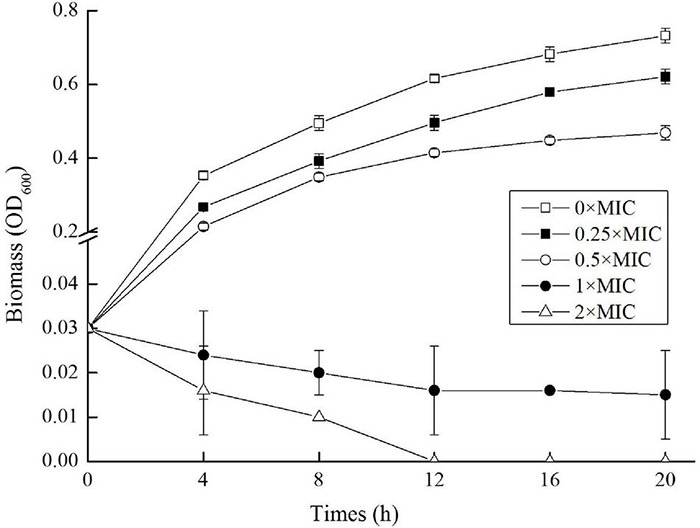
Growth curves of *Salmonella* cells exposed to different doses of chlorogenic acid (CGA).

To get the MIC value of CGA against *S. typhimurium* SL1344, OD_600_ at different incubation time was measured, and the biomass curves under different concentrations of CGA were graphed. Four representative curves (CGA doses at 12.500, 6.250, 3.125, and 1.563 mg/L, respectively) and the control (0 mg/L CGA) were drawn Figure 1. When CGA concentrations were kept at 0 mg/L (0 × MIC), 1.563 mg/L (0.25 × MIC) and 3.125 mg/L (0.5 × MIC), respectively, the growth of the *Salmonella* were inhibited as the increasing of the CGA. For example, the OD_600_ values at 20 h were sequentially 0.732 ± 0.02, 0.621 ± 0.03, and 0.469 ± 0.04, respectively. In comparison, when CGA dose was increased to 6.250 mg/L, the bacterial growth was inhibited, and the OD_600_ values decreased from the initial value to 0.015 at 20 h, thus the MIC of CGA against *Salmonella* cells at 5 × 10^5^ CFU/mL was 6.250 mg/L in the present study. When the CGA concentration reached the MIC threshold, microbial growth was significantly inhibited (*p* < 0.05). When CGA dose was further to 2 × MIC, the growth inhibitory effect was more rapidly. OD_600_ dropped to 0.01 after 4 h and approached to 0 after 12 h.

As an effective bactericidal agent for the *Salmonella*, CGA possessed high potential for application in food control and anti-infection treatment. In order to investigate the changes in characteristics of OM, CGA was added below the MIC in most instances.

### Cell Membrane Permeability

**FIGURE 2:**
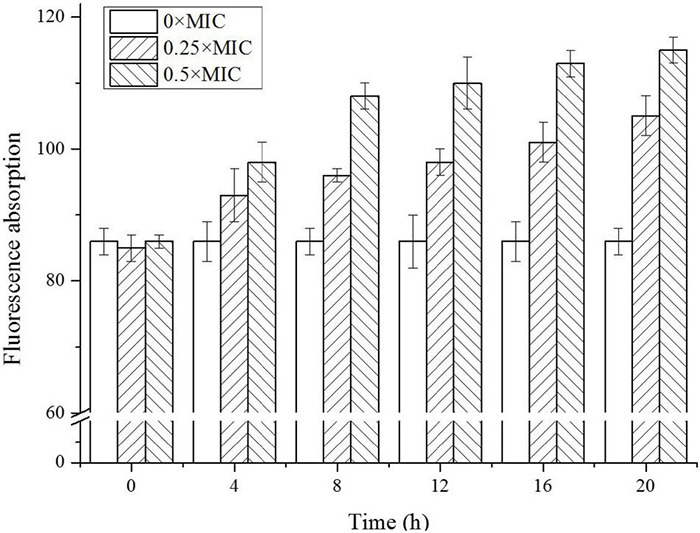
The outer membrane (OM) permeability of *Salmonella* cells exposed to different doses of CGA.

It was reported that CGA influenced the stiffness of OM (Ren et al., 2015). Since it was crucial for cell integrity and cell wall stability, the permeability of OM was studied at first. As shown in Figure 2, for the control group without CGA, the fluorescence absorbance that was positively related to permeability fluctuated slightly in the range of 85–90, suggesting that OM permeability did not change substantially. However, as the CGA dose increased, the outer cell permeability was gradually enhanced. For *Salmonella* cells treated with 0.5 × MIC, the permeability was elevated to a level approximately 30% higher than the control.

In our previous work (Wu et al., 2018b; Chu et al., 2019), the sharp increase of cell membrane permeability always led to bacterial death. The abrupt increase of OM permeability in the current study likely caused irreversible damage to OM and eventually led to rupture of bacterial cells. Therefore, the extracellular soluble proteins released outside the cells were tested.

### The Extracellular Soluble Protein Content

**FIGURE 3:**
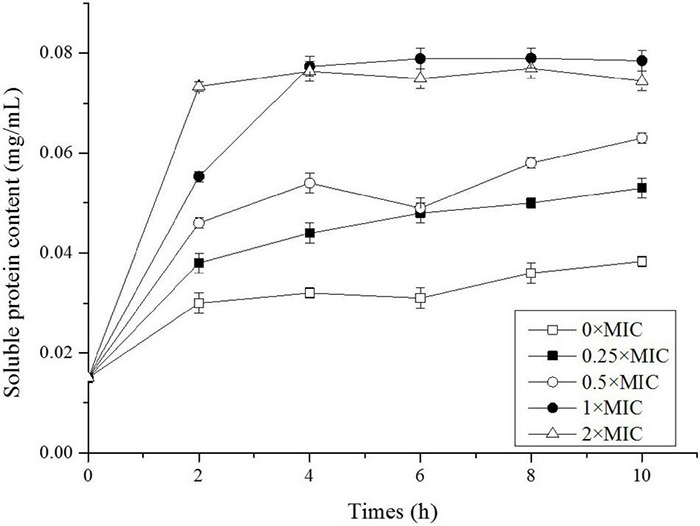
Soluble protein content of *Salmonella* exposed to different doses of CGA.

After *Salmonella* was exposed to CGA, we extracted total extracellular proteins and compared their contents (Figure 3). In the control, the soluble protein level was initially 0.015 mg/mL and slowly increased to 0.038 mg/mL at 10 h, indicating that amount of proteins normally secreted to the extracellular broth. However, when CGA was added, the soluble proteins content increased more rapidly, indicating abnormal secretion. For example, the contents under the conditions of 0.25 × MIC, 0.5 × MIC, 1 × MIC, and 2 × MIC at 2 h were 0.038, 0.046, 0.055, and 0.073 mg/mL, respectively. While for *Salmonella* cells treated by 1 × MIC and 2 × MIC for 10 h, the amounts of proteins released were close to each other, maybe because all the cells were damaged in two cases.

### Alkaline Phosphatase Activity in Bacterial Suspension

**FIGURE 4:**
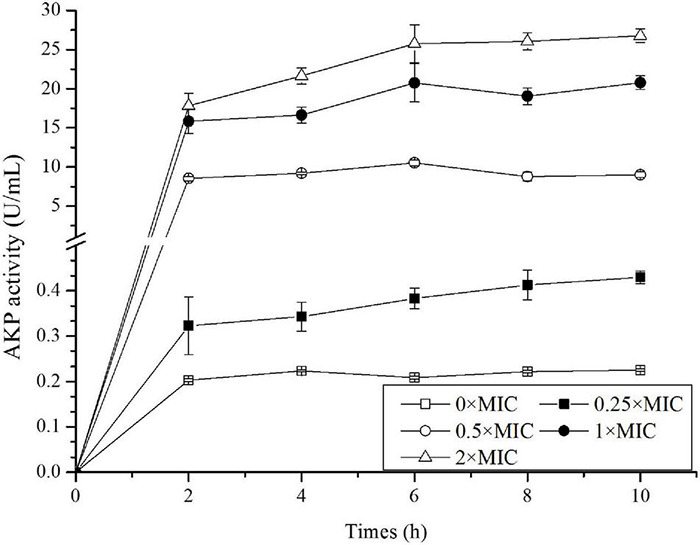
Detection of alkaline phosphatase (AKP) activity in *Salmonella* exposed to different doses of CGA.

Among the total soluble proteins released, AKP was a specific enzyme localized between OM and the inner membrane. AKP came outside of the cell in large amounts when the cell wall was damaged, thus its level in the extracellular environment was detected to assess the cellular integrity. As shown in Figure 4, AKP activity for the control was stable around 0.2 U/mL, and as expected, CGA addition stimulated APK release. The higher CGA dose, the greater APK activity. For example, APK activity for 0.25 × MIC at 10 h was approximately twice of the control, whereas APK activity for 0.5 × MIC was about 90 times greater than the control.

The results in Figures 3, 4 confirmed the leakage of intracellular contents, however, there was still a lack of morphological evidence for impairment of cell wall. Therefore, SEM was employed in the following experiment to reveal the alteration of cell surface.

### Cell Surface Morphology

**FIGURE 5:**
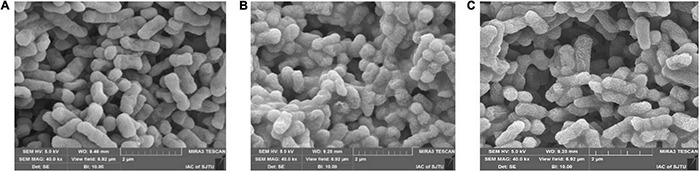
Scanning Electron Microscopy (SEM) micrographs of *Salmonella* under 0 × MIC **(A)**, 0.25 × MIC **(B)**, and 0.5 × MIC **(C)** of CGA.

The SEM photos of *Salmonella* cells treated by 0 × MIC, 0.25 × MIC, and 0.5 × MIC were shown in Figure 5. Compared to the control (Figure 5A), the samples under 0.25 × MIC CGA (Figure 5B) were more deformed and shrunken, showing evident membrane damage. Furthermore, when higher doses at 0.5 × MIC were added (Figure 5C), the most cells became atrophied, rough and wrinkled. Our results showed for the first time that CGA led to deformation of cells with additional morphological defects. All these changes easily impaired the cellular integrity. Therefore, it confirmed that CGA impaired the cellular integrity of the bacteria, and this was likely the cause of leakage of the intracellular materials and cell death.

### The Intracellular Reactive Oxygen Species

**FIGURE 6:**
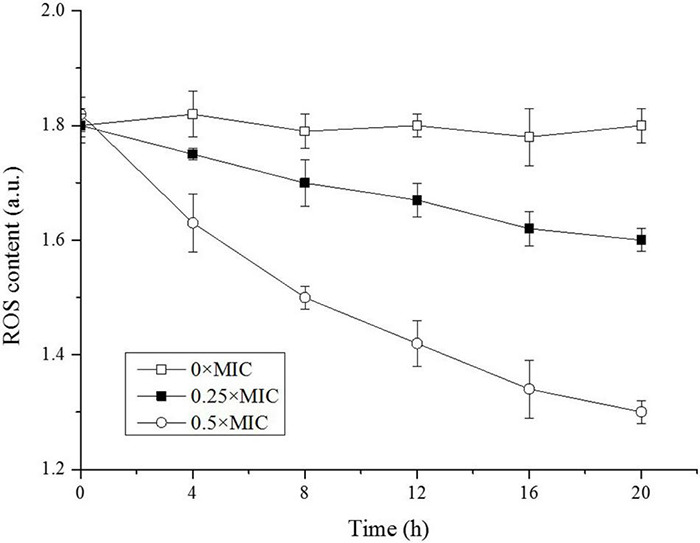
Reactive oxygen species (ROS) levels in *Salmonella* exposed to different doses of CGA.

The SEM observation confirmed damage of cell integrity induced by CGA, and the next question is how CGA impaired cell surface. There existed several possibilities, such as lipids oxidation by ROS, or changing the fluidity of OM. At first, we considered whether ROS might oxidize lipids to cause OM damage, since ROS always damaged the membranes (Hu et al., 2018; Chu et al., 2019). The intracellular ROS oxidized non-fluorescent DCFH to produce fluorescent DCF, which was detected to reflect the level of intracellular ROS (Wang et al., 2018). The time courses of the intracellular ROS were monitored and compared (Figure 6).

As shown in Figure 6, the intracellular ROS in the control was kept at 1.8 a.u. in the beginning, and remained essentially unchanged over time, indicating a balance between intracellular ROS and the antioxidant system under normal condition. However, along with the increase in CGA dose, the intracellular ROS content decreased rapidly. Unexpectedly, on the basis of these results, cell membrane damage was not associated with ROS.

### Cell Membrane Fluidity

It was widely accepted that there exist dynamical and structural changes in the membrane bilayers. The membrane fluidity was essential for making the membrane highly permeable, and abrupt change in membrane fluidity would impair the normal function of the cell membrane, and cause even bacteria survival (Learmonth, 2012). As a hydrophobic molecule, DPH can be combined with the phospholipids in the cell membrane without disturbing the structural integrity, and the degree of DPH fluorescence polarization is closely associated with the fluidity of target. An increase in membrane fluidity decreases fluorescence polarization, and vice versa (Learmonth, 2012; Choi et al., 2017).

**FIGURE 7:**
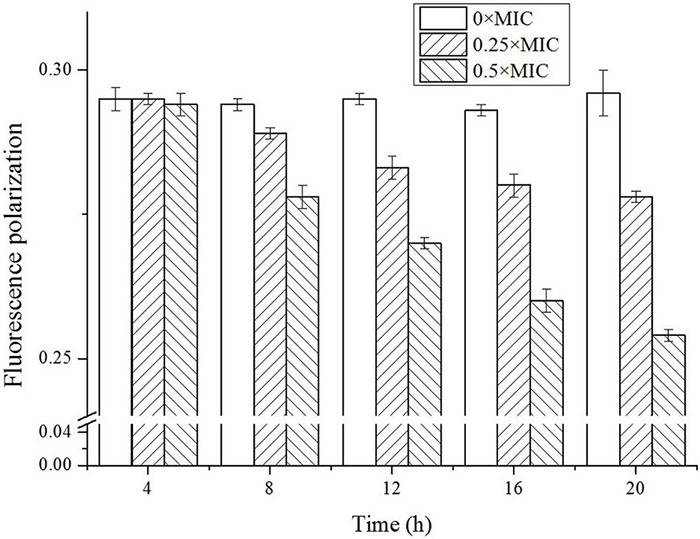
Detection of fluidity of *Salmonella* cell membrane treated by different doses of CGA.

As shown in Figure 7, the membrane fluidity showed CGA dose-dependent change. For the control without CGA addition, the fluorescence polarization was nearly stable, reflecting a high stability. However, the addition of CGA led to fluctuation of fluidity. For instance, for the cells treated with 0.5 × MIC CGA, the fluorescence polarization decreased from the initial 0.294 to the 0.254 at 20 h, reflecting an obvious rise of the cell membrane fluidity. The abrupt change of fluidity likely induced the damage of cell surface under CGA.

Taken together, the current study proved for the first time that CGA inactivated *Salmonella* cells through impairing the cellular integrity. How CGA damaged OM and increased the fluidity was an interesting question requiring further research. Analyzing the differences in the membrane compositions such as lipids will help to reveal the mechanism, and comparative lipidomics are being employed in our lab to discover the changes (Yun et al., 2016). Our study will help to better understand the bactericidal mechanism of CGA.

## Discussion

As common medicinal and edible plant food, EU had been used in China for thousands of years with health care effects, including anti-bacteria effect, while the antibacterial activity of various bioactive substances in EU, such as CGA, was reported until just recently. The present study also confirmed that at the dose beyond than 6.250 mg/L, CGA could inhibited the growth of 5 × 10^5^ CFU/mL of *Salmonella* cells in LB medium (Figure 1). *Salmonella* was regarded as one of the principal causes of foodborne outbreaks, and its pollution in dairy, egg, meat, fruit and vegetables accounted for 93.8 million cases of foodborne illness and 155,000 deaths per year (Eng et al., 2015). In addition, *Salmonella* infection led to a high death rate at 39%, greater than other foodborne pathogens. It caused a high incidence rate, for example, approximately 17.6 cases per 100,000 population in the United States (Barton Behravesh et al., 2011). Recently, the abuse of antimicrobial agents led to the emergence of multidrug-resistant *Salmonella* strains, such as *Salmonella typhimurium* DT104 (Valadez et al., 2009), it is of significance to screen the drugs targeting the essential and conservative biomolecules of bacteria, such as OM. Therefore, the current work aimed to understand the influences of CGA on the cellular integrity, and it will help to better control the food-borne pathogens, including drug-resistant *Salmonella*.

Up to now, few publications focused on the mechanisms underlying the bactericidal action. In 2015, CGA isolated from honeysuckle was reported to kill *E. coli* by influencing stiffness of OM, while the mechanism in detail was unclear (Ren et al., 2015). Besides, CGA extracted from *Arctium lappa L.* could stimulate the permeability of plasma membranes of *S. aureus* (Lou et al., 2011). The previous reports suggested that the multiple effects of CGA on OM needed to be further studied. The cell wall is a critically important entity for bacteria. The permeability of OM is essentially the ability to permit certain substances to pass in and out of the cell barrier. The membrane injury is always accompanied with permeability changes, which would lead to leakage of intracellular proteins (Yao et al., 2014; Zhang et al., 2016; Yang et al., 2020). More seriously, the increase in cell membrane permeability may lead to cell rupture and bacteria death (Jordanova et al., 2009). Our work firstly showed direct correlation between characteristics of OM (permeability, protein release, AKP release, and fluidity) and cell wall integrity (Figures 2, 3, 7). Furthermore, SEM photos provided direct evidence of the cell wall damage after CGA treatment (Figure 5). After treated by different doses of CGA, more cells had depressions, and their surfaces became rough and corrugated, thus providing direct evidence that CGA damages the cell integrity of *Salmonella*. These data will help to develop and improve novel drugs to combat antibiotic resistant bacteria (Hu et al., 2018). The cell wall is essential for their survival, and thus many drugs aimed to destroy the bacteria’s cell walls (Mayer et al., 2019). In our previous reports (Hu et al., 2018), the rupture of cell wall of microorganisms, including gram-negative bacteria, gram-positive bacteria and fungus, resulted in cell rupture very easily. The current study implied that CGA showed potential for application in anti-infection treatment.

How CGA influenced cellular integrity is still a question requiring further research. Among the possible means, the lipid oxidation by ROS is often considered a major cause of damage of the cell surface, which had been reported in our lab (Hu et al., 2018; Wu et al., 2018b; Chu et al., 2019). Therefore, change of ROS was monitored for different cells, while CGA did not stimulate but depressed ROS generation (Figure 6). This result suggested that ROS oxidation was likely not the cause of the cell membrane injury. Later, change in membrane fluidity was considered as another potential trigger for OM injury. In the preliminary experiments, other methods were also tried, with poor repeatability. Thus we employed the DPH-based method reported by Furusawa et al. (2006), achieving good repeatability.

The DPH fluorescence polarization was negatively correlated with membrane fluidity. We compared the membrane fluidity under different doses of CGA (Figure 7), and found that CGA led to an obvious increase in cell membrane fluidity in a CGA dose-dependent manner. As shown in Figure 7, the fluorescence polarization intensity of the treatment group with CGA added was significantly lower than the control, and the decrease in amplitude was greater with the addition of CGA. In the group treated with 0.5 × MIC, the fluorescence polarization decreased from an initial 0.294–0.254, indicating that the cell membrane fluidity was enhanced. A moderate cell membrane fluidity was crucial for intact cell surface, and severe fluctuations made the membrane more vulnerable to environmental stress. It was notable that the increased membrane fluidity maybe a passive result under CGA treatment, or an active response to CGA stress. In either case, it played a key role in the bactericidal process of CGA. Its exact role, either positive or negative, needed further study.

The membrane fluidity is mainly controlled by phospholipids composition (Furusawa et al., 2006). Lipids in the membrane form a classical bilayer structure, and the lipids falls into a relatively small number of general chemical classes, while the phospholipids composition and percentage are species-specific and even strain-specific (Sohlenkamp and Geiger, 2016). Different phospholipid components are assumed to contribute to specialization of function (Yao et al., 2014). In order to reveal the molecular mechanism of CGA affecting cell surface, membrane lipids compositions under CGA treatment needed further investigation. Now, the fatty acid profiles and lipidomics of the control cell and CGA-treated cells are being analyzed in our lab to reveal the global changes in lipids, and these valuable data will help to discover the mechanism of cellular integrity impairment induced by CGA.

## Data Availability Statement

The original contributions presented in the study are included in the article/supplementary material, further inquiries can be directed to the corresponding author.

## Author Contributions

LY: data curation. LY and XH: funding acquisition. LZ: investigation. CZ: methodology. ZS: software. JW: visualization. XS and XZ: writing – original draft. XH: writing – review and editing. All authors contributed to the article and approved the submitted version.

## Conflict of Interest

The authors declare that the research was conducted in the absence of any commercial or financial relationships that could be construed as a potential conflict of interest.

## Publisher’s Note

All claims expressed in this article are solely those of the authors and do not necessarily represent those of their affiliated organizations, or those of the publisher, the editors and the reviewers. Any product that may be evaluated in this article, or claim that may be made by its manufacturer, is not guaranteed or endorsed by the publisher.
